# Quantum uncertainty switches on or off the error-disturbance tradeoff

**DOI:** 10.1038/srep26798

**Published:** 2016-06-01

**Authors:** Yu-Xiang Zhang, Zu-En Su, Xuanmin Zhu, Shengjun Wu, Zeng-Bing Chen

**Affiliations:** 1 Kuang Yaming Honors School and Department of Physics, Nanjing University, Nanjing, Jiangsu 210023, China; 2 Hefei National Laboratory for Physical Sciences at Microscale, The CAS Center for Excellence in QIQP and the Synergetic Innovation Center for QIQP; Department of Modern Physics, University of Science and Technology of China, Hefei, Anhui 230026, China; 3 School of Physics and Optoelectronic Engineering, Xidian University, Xi’an 710071, China

## Abstract

The indeterminacy of quantum mechanics was originally presented by Heisenberg through the tradeoff between the measuring error of the observable A and the consequential disturbance to the value of another observable B. This tradeoff now has become a popular interpretation of the uncertainty principle. However, the historic idea has never been exactly formulated previously and is recently called into question. A theory built upon operational and state-relevant definitions of error and disturbance is called for to rigorously reexamine the relationship. Here by putting forward such natural definitions, we demonstrate both theoretically and experimentally that there is no tradeoff if the outcome of measuring B is more uncertain than that of A. Otherwise, the tradeoff will be switched on and well characterized by the Jensen-Shannon divergence. Our results reveal the hidden effect of the uncertain nature possessed by the measured state, and conclude that the state-relevant relation between error and disturbance is not almosteverywhere a tradeoff as people usually believe.

Quantum uncertainty is conventionally explained through two sequential quantum measurements^1^,^2^, the error of the first and its disturbance to the precision of the second are qualitatively claimed to have an essential tradeoff. The core effect in the relation between error and disturbance is the intrinsic back action of quantum measurements, as Dirac wrote “*a measurement always causes the system to jump into an eigenstate of the dynamical variable that is being measured* 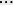
”^3^. Heisenberg only gave an intuitive and informal argument of the *probable* error-disturbance tradeoff. The famous uncertainty inequalities, Kennard’s^4^ Δ_*x*_Δ_*p*_ ≥ *ħ*/2 (Δ is the standard deviation), Robertson’s^5^ 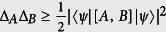 and Maassen-Uffink’s^6^,^7^,^8^,^9^ entropic inequality all focus on limitations of legal states, but do not cover the characteristic back-action of quantum measurements.

To close the discrepancy between mathematical inequalities and physical interpretations, Ozawa firstly quantified the error and the disturbance, and analyzed their relationship. He proposed an inequality^10^:


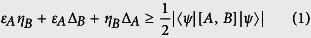


and claimed the incorrectness of the Heisenberg-type tradeoff *ε*_*A*_*η*_*B*_ ≥ |〈*ψ*|[*A*, *B*]|*ψ*〉|/2. There in, the error term, *ε*_*A*_, is defined as the root mean squared of the difference between the observable really measured and *A*, the observable we aimed at. The disturbance to *B*, *η*_*B*_, is defined in a similar manner. We shall not repeat the expressions of *ε*_*A*_ and *η*_*B*_ but emphasize that they are defined as being *state-relevant*, i.e., they are defined for each specific input state |*ψ*〉.

The mathematical correctness of Eq. (1) has been verified extensively in qubit experiments^11^,^12^,^13^,^14^,^15^,^16^. However, its physical exactness was questioned is triggering a heated debate on the error-disturbance tradeoff^17^,^18^,^19^,^20^,^21^,^22^,^23^,^24^,^25^,^26^,^27^. The *ε*_*A*_ and *η*_*B*_ defined by Ozawa were criticized for violating the *operational requirements* for exact and faithful definitions of error and disturbance, which are explicitly suggested in ref. 27. They require

- error to be nonzero if the outcome distribution produced in an actual measurement of *A* deviates from that predicted by Born’s rule;
- disturbance to be nonzero if the back-action introduced by the actual measurement alters the original distribution with respect to *B*.

These requirements emphasize the probabilistic feature of quantum mechanics, and further imply that 〈*ψ*|[*A*, *B*]|*ψ*〉 should be excluded to appear alone on the right hand side of the inequalities that describe the tradeoff^27^. This conclusion sharply conflicts with Eq. (1) and leaves an open problem as what can be there.

Meanwhile, Busch, Lahti and Werner (BLW) developed theories to support Heisenberg-type relations^24^,^25^. In their inequalities, the relevance to states is erased by searching the extreme case over all possible inputs. The relevance to input state has not been well taken into account^26^. These theories behave like benchmarking the quality of the measurement device. Unlike those state-relevant formalisms, they cannot describe the tradeoff in each particular experiment.

In spite of the intricate features, we notice the presence of Δ_*A*_ and Δ_*B*_, characters of the quantum uncertainty, in Eq. (1). It inspires us to ask the question: does the *quantum uncertainty* possessed by the quantum state with respect to *A* and *B* play some hidden but intrinsic role in the error-disturbance tradeoff? Meanwhile, those state-irrelevant inequalities hint that the state-relevant relation may not be a pure tradeoff. In this paper, we will propose a state-relevant formalism of the error-disturbance relation with definitions of the error and the disturbance that obey the operational requirements. We demonstrate both theoretically and experimentally that a condition controlled by quantum uncertainty determines the existence of the error-disturbance tradeoff. We also give an inequality for the case where the tradeoff does exist.

## Results

### Error and disturbance

**Figure 1:**
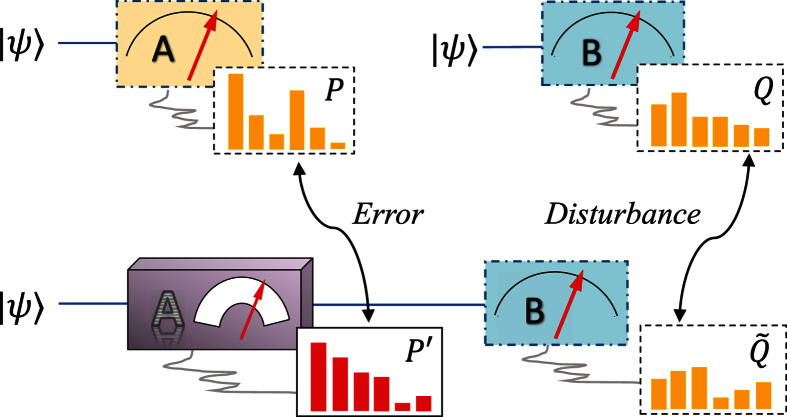
Error and Disturbance. Boxes with dashed frames stand for the ideal measurements with the outcomes given by Born’s rule. The other box stands for the real-life apparatus which produces the distribution *P*′. Error is defined by the divergence between *P* and *P*′. The difference between *Q* and 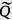 witnesses the disturbance caused by the inevitable back-action by the first measurement. Thus we define the disturbance by comparing *Q* and 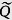.

Suppose we measure observable *A* on a quantum system in state |*ψ*〉 with an imperfect real-life device. In the process, the employed device (together with the environment) selects a preferred pointer basis in the system’s Hilbert space^28^. As a result, the entire state evolves to be 
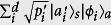
, where indexes *s* and *a* label the system and the apparatus (also the environment), and 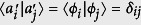. The apparatus bridges the quantum system and the classical world we stay in. It returns an outcome from the set 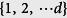. Repetitive measurements generate the distribution 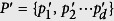 with 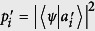. By the logic of quantum mechanics, what we learn from quantum measurements is just this distribution. However, due to kinds of imperfections, 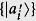 is hard to be exactly the eigenstates of *A*, {|*a*_*i*_〉}. This mismatch, i.e., the error, is therefore embodied in the difference between *P*′ and 
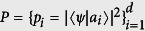
, the distribution predicted theoretically by Born’s rule. Thus, we define the error, *Err*_*ψ*_(*A*), to be specified below, to quantify the difference between *P* and *P*′ (see Fig. 1).

By the notation “|*ψ*〉” and the specified process of measurements, we have assumed that the problem in consideration associates with pure-state systems and the projective measurements that are maximally informative^29^. In fact, all the quantum measurements can be modeled by tracing back to projective measurements performed on a complete system described by a pure state. The concepts of mixed states and positive-operator valued measurements (POVM) emerge by statistically ignoring some sub-systems and losing some classical information. We will discuss them at the end and here focus on the most underlying structure.

Now we define the disturbance. Statistically, the back-action of the real-life measurement maps the original state |*ψ*〉 to 
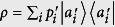
. This is the disturbance. When referring to observable *B*, it means that the original distribution determined by Born’s rule, 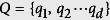 with 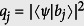, is disturbed to 
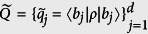
. Therein, {|*b*_*j*_〉} are eigenstates of *B*. If 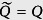, the disturbance to the quantum state induced by the real-life measurement of *A* cannot be perceived from observing *B*. So we define the disturbance to *B*, *Dis*_*ψ*_(*B*), by the divergence between 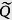 and *Q*.

**Figure 2:**
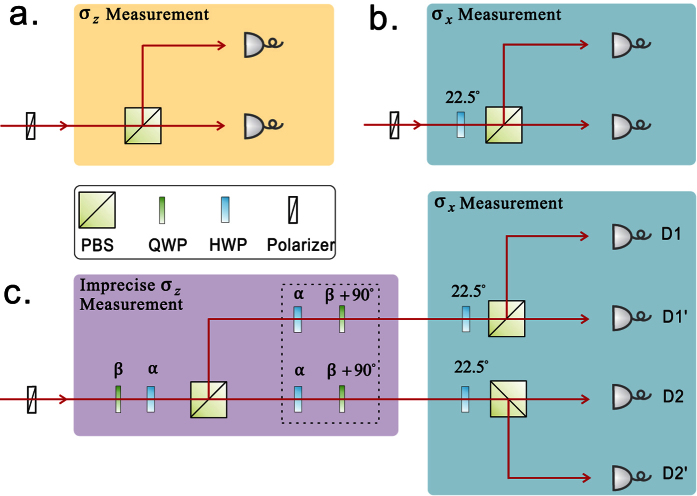
Experimental setup. Here part (**a**) and part (**b**) show the setup for ideal measurement of *A* = *σ*_*z*_ and *B* = *σ*_*x*_ respectively. Part **c** shows the sequential measurements of *A* (imprecise) and *B*. The groups of half-wave plate and quarter-wave plate positioned behind the PBS (in the box with dashed frame) rotate |*H*〉, |*V*〉 to the eigenstates of 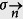, the observable actually measured in the imprecise measurement. A polarizer is used to prepare the initial state of the photons. Finally, the photons are sent to fiber-coupled single-photon avalanche photodiode detectors.

It can be seen that our definitions satisfy the operational requirements introduced above in a *minimal* way. That is, our definitions satisfy no additional requirements other than the operational requirements. Furthermore, the operational definitions will offer us a direct experimental implementation as illustrated in Fig. 2. With these preparations, we can now give one of the main results.

### Quantum uncertainty and error-disturbance tradeoff

We find that a criterion for which quantum uncertainty completely determines the existence of the state-relevant error-disturbance tradeoff.

**Theorem 1.** *For any d-dimensional pure state* |*ψ*〉, *there exists projective measurements such that Err*_*ψ*_(*A*) *and Dis*_*ψ*_(*B*) *vanish simultaneously, i.e., there is no tradeoff between the error of measuring A and the consequential disturbance to B*, *if and only if* 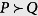.


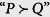
, read *P* majorizes *Q*, means that if sorting the elements from large to small, i.e., 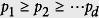 and 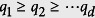, then 
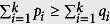
 for 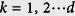
30. That means the occurrence probability of a top-*k* high-probability outcome in the ideal measurement of *A* is no less than that of *B*, for all possible values of *k*. Thus, majorization gives a rigorous criterion for the partial order of certainty or uncertainty. Moreover, 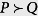 leads to *H*(*P*) ≤ *H*(*Q*), where 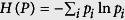 is the Shannon entropy. As widely accepted, larger Shannon entropy means more uncertainty. So Theorem-1 concludes that, if the outcome of measuring A is more certain than the outcome of measuring B, there will be no state-relevant error-disturbance tradeoff; otherwise, the tradeoff will be switched on and a positive lower bound of *Err*_*ψ*_(*A*) + *Dis*_*ψ*_(*B*) is expected.

The necessary part of Theorem 1 can be derived from a historic mathematical theorem^31^. The necessary part says if P and Q are generated in sequential measurements, P must majorize Q. That is, measurement is a way of extracting information thus the entropy is generally increasing. The sufficient part is more technic and we leave the proof in Methods. Our proof sets up an algorithm to find generally 2^*d*−1^ different realizations of 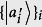 to close the tradeoff. There are two known extreme examples where there is no tradeoff. One is the case where |*ψ*〉 is an eigenstate of *A*. Another case is the zero-noise and zero-disturbance (ZNZD) states defined in ref. 27. In the first example of eigenstates, *P* = {1, 0, … 0}; for ZNZD states, *P* and *Q* are both the uniform distribution 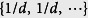. So *P* majorizes *Q* in both cases. Here by revealing the effect of quantum uncertainty behind the scene, Theorem 1 links these isolated point-like examples together and extends the no tradeoff conclusion to extensive situations.

Theorem 1 does not conflict with the common sense that non-commuting observables are impossible to be precisely measured simultaneously with a state-independent strategy, because the vanishing tradeoff between error and disturbance implied by Theorem 1 is conditioned on the input state. An inverse question may be interested: given the real life measurements along 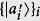, how many input states share the merits of zero-error and zero-disturbance? We leave it to the Discussion.

### Quantification of the tradeoff

If 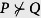, the error-disturbance tradeoff is switched on. To describe the tradeoff, we shall quantify *Err*_*ψ*_(*A*) and *Dis*_*ψ*_(*B*). Based on the above analysis, we can quantify them using any non-negative functional *D*(·, ·) that vanishes only when the associated two probability distributions are identical. The relative entropy 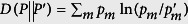, a central concept in information theory with wide applications, is one such example. Explicitly, we quantify the error and the disturbance as


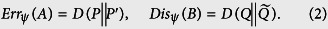


These information-theoretical definitions are independent of the unessential eigenvalues of the relevant ovservables, like the entropic uncertainty relation^6^,^7^. This is different from the Wasserstein 2-deviation used in the works of BLW. In the following, we give the result for 2-dimensional cases at first.

**Theorem 2.** *When d* = 2 *and* 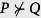
*, let us label the outcomes so that p*_1_ ≥ *p*_2_ *and q*_1_ ≥ *q*_2_ *and quantify error and disturbance by the relative entropy as Err*_*ψ*_(*A*) = *D*(*P* ‖ *P*′) *and* 
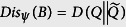
*. Then we have*


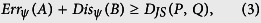


*where the Jensen-Shannon divergence*, 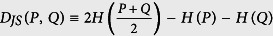 (*H is the Shannon entropy*), 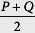 *is the distribution* 
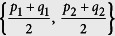
.

**Figure 3:**
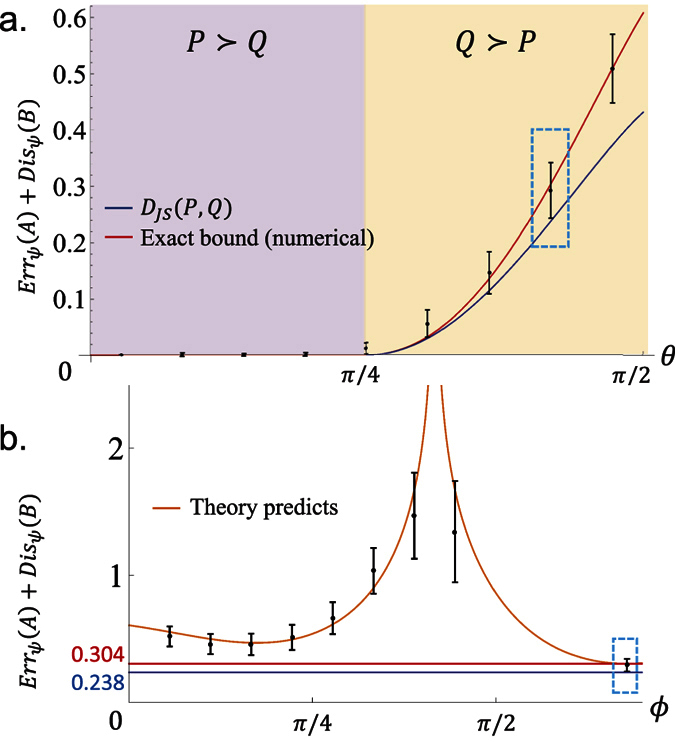
Experimental results. In part (**a**) the red line is the exact lower bound of *Err*_*ψ*_(*A*) + *Dis*_*ψ*_(*B*) which we obtain via numerical methods. The blue line is the lower bound given by *D*_*JS*_(*P*, *Q*) in Theorem 2, it can be very close to the exact bound given in the red line. In part (**b**) as a function of *ϕ*, *Err*_*ψ*_(*A*) + *Dis*_*ψ*_(*B*) is always higher than the bound given by 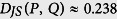. The point marked by light blue dashed rectangle reaches the minima value of 0.304 that is obtained numerically, it is identical to the point marked in part (**a**). The error bars indicate the standard deviation including both systematical (±1° rotation of each plate) and statistical errors (Poissonian distribution).

In Fig. 3, we illustrate both *D*_*JS*_(*P*, *Q*) and the exact bound obtained by numerical calculations. *D*_*JS*_(*P*, *Q*) is shown to be a valid lower bound which can be very close to the exact one. Theorem-2 answers the open question asked in ref. 27 of what can serve on the right hand side of the state-relevant inequality. The Jensen-Shannon divergence is also a distance function of probability distributions that has been applied in bioinformatics, machine learning and social science^32^. Interestingly, the well-known Holevo’s bound^33^, the upper bound to the accessible information of a quantum state, is its quantum generalization.

Next we shall give the strategy for constructing the inequalities, and then generalize Theorem 2 to the case of higher dimensions.

#### Strategy for the lower bound

**Figure 4:**
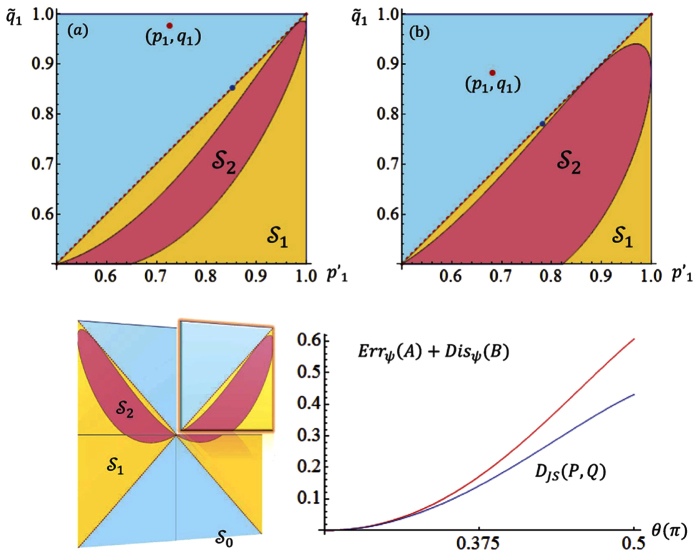
Two-dimensional case: imbedding 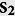 and 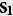 in 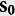 when *d* = 2. 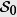
 is parameterized by 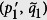 and thus illustrated by the [0, 1] × [0, 1] square. We fix the labeling of eigenstates of *A* and *B* such that *p*_1_ ≥ *p*_2_ and *q*_1_ ≥ *q*_2_. 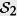 is the pink region, it is a subset of 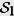 that is contained in the yellow region. When the relative entropy is used to characterize the distance of probability distributions, it is sufficient to consider only the component with 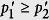 and 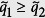. (a,b) show the details of that component when 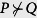 and (*p*_1_, *q*_1_) equals to (0.727, 0.978) and (0.681, 0.882), respectively. The lower bound *D*_*JS*_(*P*, *Q*) is obtained on the blue points, which locate on the surface of 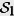. The line-chart illustrates the exact lower bound (red) of *Err*_*ψ*_(*A*) + *Dis*_*ψ*_(*B*) and lower bound *D*_*JS*_(*P*, *Q*) (blue) determined by Theorem-2, when *A* = *σ*_*z*_, *B* = *σ*_*x*_, and 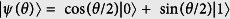 with 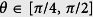 such that 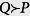. Here, 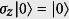 and 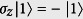.

As coordinates, all pairs 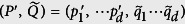 as defined above compose a set we call 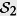. We can release the specified definitions to view *P*′ and 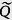 as free distributions. Then the pairs 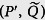 compose a 2(*d* − 1)-dimensional sub-manifold 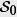 of the 2*d*-dimensional Euclid space (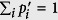 and 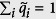). The exact bound of *Err*_*ψ*_(*A*) + *Dis*_*ψ*_(*B*) is the minima of 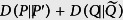 over 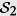. An analytical solution seems complicated to approach and shapes terrible because of the involved geometry of 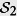 embedding in 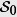. Instead, we define the set of pairs satisfying 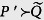 as 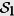. According to Horn’s theorem^30^, we have 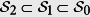 and thus 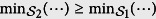 by logic. Moreover, the point 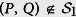 is the unique extreme point of *Err*_*ψ*_(*A*) + *Dis*_*ψ*_(*B*) in 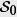 such that its minima value over 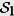 must be obtained at the surface of 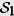. This surface consists of many faces, especially those defined by majorization. The geometry of 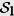 is much simpler and analytical solution becomes reachable. So the strategy is to find the minima over 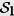. An illustration of this strategy for the case d = 2 is given in Fig. 4. The strategy also works for many other quantifications of the difference between probability distributions, aside from relative entropy.

Physically speaking, the strategy is equivalent to replacing the ideal measurements of *B* (in the part of sequential measurements in Fig. 1) with an imprecise apparatus performing projective measurements in a random basis denoted by 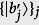. Now 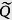 should be redefined as 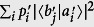. With the help of Horn’s theorem^30^, the exact lower bound of 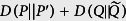 is just the minima of the original *Err*_*ψ*_(*A*) + *Dis*_*ψ*_(*B*) over 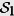.

On the way to the bound, a new concept emerges as a natural extension of majorization.

#### Majorization by sections

If 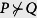, we cannot conclude that *P* is more certain than *Q* in the global sense. However if only certain outcomes are considered, things will be different. Let us relabel the eigenstates so that 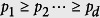 and 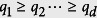, then cut the subscript string 1 ~ *d* into short sections 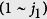, 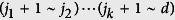. For each section, say the *t*-th one, we find the probabilities according to the subscripts in this section and take their sum 
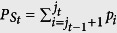
 and 
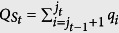
 (*j*_0_ = 1, *j*_*k*+1_ = *d*). Then we say *P majorizes Q by sections* if the relation


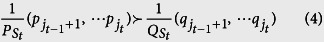


holds for all the short sections. (If some zero-valued probabilities vanish the denominator, take the limit from infinitesimal positive factors). Equation (4) says that *P* is relatively more certain than *Q* in each section. We use 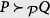 to denote this relation where the index 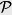 labels this particular partition of the subscript string. In addition, 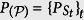 and 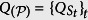 are two distributions coarse-grained from *P* and *Q* by this partition.

Next, let us find all the coarsest partitions under which *P* majorizes *Q* by sections. We say a partition is coarser than another if the latter can be obtained from the former by additional cutting such as cutting (2 ~ 9) into (2 ~ 4) (5 ~ 9). (“ Coarser” defined in such a way is a partial order, we cannot say (1 ~ 5) (5 ~ 9) is coarser than (1 ~ 4) (4 ~ 7) (7 ~ 9)). Let us denote the set of all the coarsest partitions upon which *P* majorizes *Q* by sections as 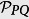. None of 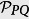 can be obtained by further cutting from another section in it. 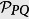 is never an empty set since *P* will always majorize *Q* by sections for the finest partition where each short section consists of only one subscript. Then according to each partition in 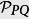 (say, the one labeled by 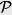), we coarsen *P* and *Q* to obtain distributions 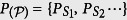 and 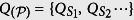 in the way given above. With these preparations, now we can present our tradeoff relation.

**Theorem 3.** *Label the outcomes such that* 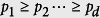 *and* 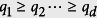
*. Then if* 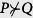
*, Err*_*ψ*_(*A*) = *D*(*P* ‖ *P*′) *and* 
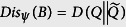
*, there is a tradeoff relation*


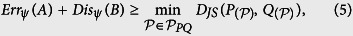


*where* 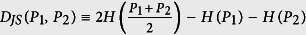 *is the Jensen-Shannon divergence. Moreover*, *D*_*JS*_(*P*, *Q*) *serves as the lower bound if* 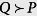 *and* 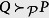 *for any possible partition* 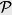.

## Experiment

Being based on operational definitions, our theory can be experimentally tested in a straightforward manner. To compare, the reported experimental tests of Ozawa’s inequality (qubit cases) require the three state method or the technology of weak measurement^11^,^12^,^13^,^14^,^15^,^16^. These experimental configurations are out of the original physical picture thus not so direct^34^.

For single-photon experiments, a weak coherent state measured by single photon detectors is a good approximation to a true single-photon source^35^. Thus, as a photon source, we used a pulsed laser (with 788-nm central wavelength, 120-fs pulse width and 76-MHz repetition rate; Coherent Verdi-18/Mira-900F) and highly attenuate its mean photon number to ~0.004 at each pulse. For measurements, we can measure *σ*_*z*_ by two single-photon detectors after the polarizing beam splitter (PBS, extinction ratio >500), which transmits horizontal and reflects vertical polarizations (Fig. 2a). The *σ*_*x*_ measurement, which corresponds to the polarization measurement in the ±45° linear polarization basis, could be realized by inserting a half-wave plate (HWP) rotated at 22.5° before the PBS (Fig. 2b). The real-life measurements of *A* are actually measuring observables 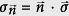, the direction 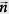 is a unit vector. They are realized by a PBS which performs *σ*_*z*_ measurement, and groups of HWP and quarter-wave plate (QWP) that implement unitary rotations between the basis {|*H*〉, |*V*〉} and the eigenbasis of 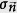 (Fig. 2c). The ratio of the counts of D1 + D1’ and the total counts determines 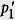, and 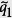 is determined by the ratio of the counts of D1’ + D2 and the total counts. The distributions *P*′ and 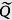 are thus obtained.

In the experiment, a polarizer was used to prepare the photons in the state 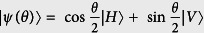 with 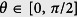. Therein, |*H*〉 and |*V*〉, the horizontal and vertical polarization states, are viewed as the eigenstates of *σ*_*z*_, i.e., *σ*_*z*_|*H*〉 = |*H*〉 and *σ*_*z*_|*V*〉 = −|*V*〉. We calculated the optimal directions 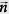 so that the measurements of 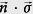 could reach the minimal sum of *Err*_*ψ*_(*A*) and *Dis*_*ψ*_(*B*), where 
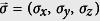
. These directions are used to carry out the imprecise measurements of *A* of which the experimental results are illustrated in Fig. 3a. Theoretically, we have 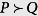 when 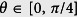 and 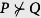 when 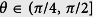. So there is no tradeoff in the first half *θ*-range by Theorem 1 and Theorem 2 covers the rest. Experimental results show great agreements with the predictions of the two theorems (see Fig. 3a, where the closeness of *D*_*JS*_(*P*, *Q*) is illustrated by comparing with the exact bound obtained by numerical calculations). The validity of the bound given by Theorem 2 is also tested with a randomly chosen state with parameter *θ* = 5/12*π*(75°). The real-life measurements of *A* are implemented by the measurements of 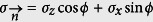. Thus *Err*_*ψ*_(*A*) + *Dis*_*ψ*_(*B*) varies as a function of *ϕ*. As shown in Fig. 3b, experimental data clearly supports the theory.

## Discussion

If we focus on a sub-system of a pure-state system, mixed input states must be taken into account. Now actually classical uncertainty is involved in. We show in the Methods that Theorem 2 and Theorem 3 are valid for all the mixed input states, and Theorem 1 is robust to depolarization noise, i.e., valid for at least the ensembles described by 
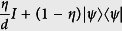
 with 0 ≤ *η* ≤ 1), as well as for all qubit states, pure or mixed. The validity of Theorem 1 in more general situations is still an open question. Additionally, we considered only projective measurements. The more general POVMs are realized from projective measurements on the enlarged systems. Correlation between the measured system and the ancillary established in the implementation of POVM, and the consequent information flow between them make the problem more complicated^27^. The state-dependent relation between error and disturbance thus still have rich structures to be discovered.

For some state |*ψ*〉, if 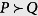 we can find 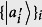 so that *Err*_*ψ*_(*A*) = *Dis*_*ψ*_(*B*) = 0. While, given this 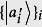, how many input states, mixed or pure, ensure zero-error and zero-disturbance? The *d*-dimensional quantum state *ρ* can be parameterized using a vector 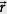 so that


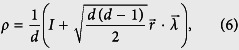


where the generators of the Lie-algebra of group *SU* (*d*), 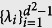, satisfy tr(*λ*_*i*_*λ*_*j*_) = 2*δ*_*ij*_. 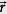 should satisfy some conditions to make *ρ* positive. 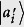, |*a*_*i*_〉 and |*b*_*j*_〉 can also be represented in the same way (by 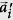, 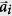, and 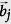, respectively). Then zero-error and zero-disturbance, i.e., 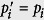 and 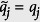, are described by


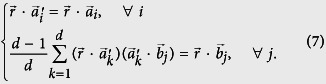


This series contains 2(*d* − 1) independent equations while 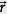 has *d*^2^ − 1 coordinates. Therefore, the solution set of 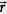 has at most (*d* − 1)^2^ dimensions, and includes at least |*ψ*〉 and the mixed states 
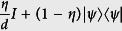
.

To conclude, we have revealed the hidden effect of quantum uncertainty on the error-disturbance tradeoff. Our results also shed new light on overcoming problems engendered by the back-action of quantum measurements in fields such as quantum control, quantum metrology and measurement-based quantum information protocols^36^. We hope this work can inspire more research on quantum uncertainty and measurement.

## Methods

**Proof of theorem 1.** The proposition that error and disturbance can be zero simultaneously is equivalent to the proposition that there is a unitary matrix which satisfies the following two conditions simultaneously:


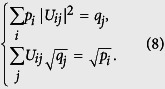


Here we just need to show sufficiency. For *B*, we have the freedom to define the phases of its eigenstates 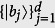 so that the state |*ψ*〉 can be written as


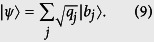


Then if 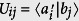 satisfies the above two condition, we have


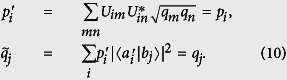


Then we will prove the existence of such a unitary with the premise 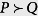 by mathematical induction. (Horn’s theorem states that the first condition has solutions if and only if 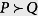). First, when *d* = 2, if 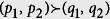 (for convenience, we assume *p*_1_ > *p*_2_, the case *p*_1_ = *p*_2_ is trivial), the following unitary results


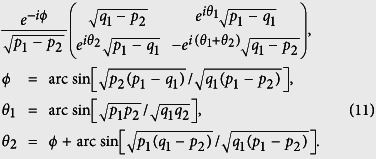


Actually, we will get a second solution by taking −*ϕ*, −*θ*_1_ and −*θ*_2_ in the above matrix. Here, we do not require the normalization that 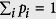. Then we assume the validity of the cases where the dimension equals to *d* − 1.

For *d*-dimensional cases, the first condition can be written as


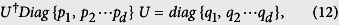


where we use *Diag* to denote the diagonal matrix and 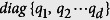 means a matrix whose diagonal elements are 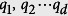.

For convenience, we assume that 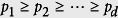 and *q*_1_ is the largest one in *Q*. There exists a subscript *j* such that


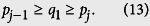


Then we have


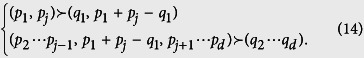


For the first, majorization is valid since 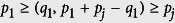. For the second majorization, when 2 ≤ *k* ≤ *j* − 1, since 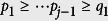, we have 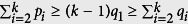; when *j* ≤ *k* ≤ *d* − 1, we have 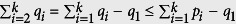. Since 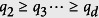, the second majorization must be valid.

Then we have a unitary *U*_1_ that acts only on the subspace belonging to *p*_1_, *p*_*j*_, such that it changes the diagonal elements *p*_1_, *p*_*j*_ to *q*_1_, *p*_1_ + *p*_*j*_ − *q*_1_ and maps vector 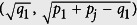. Next, according to our induction assumption, we have another unitary *U*_2_ acting on the subspace belonging to *p*_2,_ 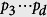 that changes *Diag* 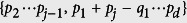 to 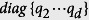 and maps vector 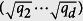 to 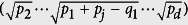. Then *U*_1_*U*_2_ is the unitary we want for the *d*-dimensional cases.

Since we have two solutions when *d* = 2, it can be seen from the induction that generally 2^*d*−1^ solutions can be found.

**Proof of Theorems 2 and 3.** Theorem 2 is covered by Theorem 3, thus we give only the proof of the latter. For convenience, we make use of the freedom of relabeling to assume that 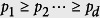 and the same applies for distributions *Q*. Then *P*′ and 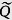, which are also labeled by such an order, will give 
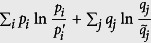
 the smallest value.

*Lemma.* If the probability distributions *P* and *Q* are sorted by the order that 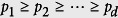 and 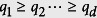, then among all ways of labeling the probabilities in *P*′ and 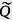, the one satisfying 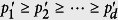 and 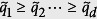 gives the minima to 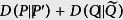. The proof is omitted here.

Without loss of generality, we assume that elements in *P* and *Q* are all positive. For possible problems caused by zero elements, we can take the limit from infinitesimal positive factors. Consider the geometric surface of 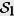 in manifold 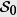. First, it is composed by (*d*!)^2^ symmetric components due to permutation. The above lemma tells us that we only need to consider the single component on which *P*′ and 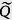 are labeled in decreasing order. Such a component looks like a polytope with many faces and we only need to take the faces associated with the definition of majorization into account. (Faces associated with equations like 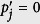 or 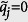 points on them will give infinite value to the sum of error and disturbance, thus we do not need to care about them; other faces of the component associated with the decreasing order of labeling are not faces of 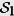). Consider the following equations


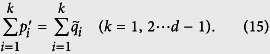


Now we use *n* to denote the dimension of the manifold, i.e., *n* = 2(*d* − 1). An (*n* − *j*)-dimension surfaces of 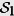 is produced by *j* equations of the above equation string, accompanied with the restriction that 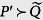.

Now let us consider the minimum value on an (*n* − *k*)-dimension surface (*j* ranges from 1 to *d* − 1). The *j* equations cut the subscript string 1 ~ *d* into *k* + 1 sections that the sum of 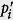 and the sum of 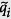 within each subscript-section are equal. We use *S*_*t*_ to denote the different sections and define notations


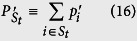


do the same for the distributions *P*, *Q* and 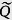. Then we use the Lagrange multiplier method to search for the extreme value:


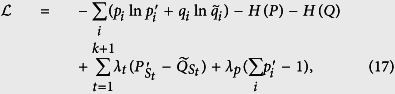


where the equivalence in each section has already implies that 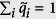. Simple calculation shows that the minima is obtained on the point


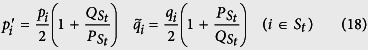


if the subscript “*i*” is in the *t*-th section where *λ*_*p*_ = 2 and 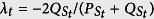. To write down the minimum value, we define two distributions obtained from *P*, *Q* by coarse graining:


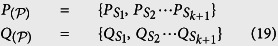


and an average of the two, 
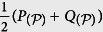
 with elements 
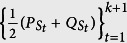
. Then the minimum value obtained from Eq. (18) is given by the Jensen-Shannon divergence, 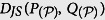.

Now we have to check whether this point is located on the (*n* − *j*)-surface of 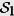, i.e., whether 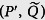 given by Eq. (18) satisfies the requirement that 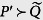. Since 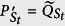, 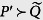 if and only if *P* and *Q* satisfy the condition that within any section, such as *S*_*t*_. After re-normalizing 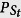 and 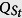 to a common factor we must have 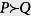 in each section. More rigorously, suppose that section *S*_*t*_ has subscripts 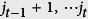, then 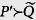 if and only if Eq. (4) is valid for all these sections. This is the conception of “majorization by sections” introduced above.

If the above condition is not satisfied, the point defined by Eq. (18) locates outside of 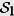 so we should consider the edges of the (*n* − *j*)-dimensional surface, i.e., we should add another equation in Eq. (15) and study the (*n* − *j* − 1)-dimensional case. If the above condition is satisfied, a finer partition, i.e., adding extra equations in Eq. (15), will not bring a lower value.

So we have to find the family of all the coarsest partitions of the subscript string (anyone in this family is not a refinement of another one in it) under which *P* majorizes *Q* by sections. Then we calculate the Jensen-Shannon divergence corresponding to the partitions and the minimum is just the minimum over 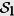. With the notations 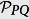, the above analysis leads to Theorem 3.

One may wonder whether the solution given by Eq. (18) follows the order 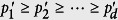 and 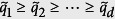. Actually, we do not need to care. This is because 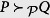 ensures 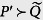 such that the solution is in 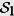. Thus all the values derived from 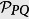 can be reached in 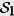, and meanwhile the real minima over 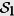 must link with one partition in 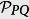. So the minimizing over 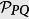 will always give the minima we want.

The second part of Theorem 3, which gives the sufficient condition for the situations when *D*_*JS*_(*P*, *Q*) serves as the lower bound, can be checked straightforwardly. Theorem 2 is covered in this case.

### Generalization to mixed input states

Mixed states emerge by statistically ignoring some sub-systems and losing some classical information, i.e., including classical uncertainty in the scenario. Theorem 3, inequality for the tradeoff, is still valid because the relation 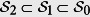 holds for mixed input states. Actually one can do more analysis in 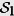 to get a tighter bound, since *P*′ is also majorized by the string of eigenvalues of the input *ρ*. Other versions of Theorem 3 could be derived with other metrics of distance between probability distributions. The relative entropy could be infinity. One with finite upper bound can be utilized if someone wanted to derive a BLW-type theory from ours.

As to Theorem 1, let us give it another proof for qubits. The density matrix of the input state, *A*, *B* and the observable of the real-life measurement *O*_*A*_, can be represented by four vectors 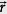, 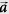, 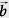 and 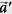 in Bloch sphere. The probability distributions have one to one correspondence with the inner products such as 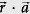 and 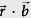. They can be assumed to be positive due to the freedom of relabeling the eigenstates of *A* and *B*. Suppose the angle between 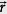 and 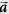 is *θ*_*a*_ and the angle between 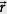 and 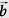 is *θ*_*b*_. Now 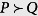 implies that *θ*_*a*_ ≤ *θ*_*b*_. 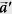 can be obtained by rotating 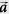 around 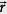 such that *P*′ = *P*. Thus the angle 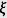 between 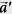 and 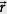 will range from *θ*_*b*_ − *θ*_*a*_ to *θ*_*a*_ + *θ*_*b*_. Then there must be a case where we have 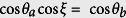, which then leads to 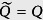. It can be seen from the proof that what important is not the norm of 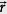, but rather its direction and the inter-angles between the vectors representing |*ψ*〉, 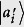 and |*b*_*j*_〉.

For higher dimensions, projectors of pure states can also be represented by coherent vectors as


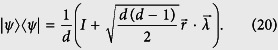


We have proved Theorem 1 for pure states. Drawing an analogy with the qubit case, we conclude that Theorem 1 is valid for mixed states in the form of 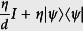, which have parallel but shorter coherent vectors compared with |*ψ*〉. In other words, Theorem 1 is robust under depolarization de-coherence. The validity of Theorem 1 for more general mixed states is still open.

## Additional Information

**How to cite this article**: Zhang, Y.-X. *et al.* Quantum uncertainty switches on or off the error-disturbance tradeoff. *Sci. Rep.* **6**, 26798; doi: 10.1038/srep26798 (2016).
